# The coming Omicron waves and factors affecting its spread after China reopening borders

**DOI:** 10.1186/s12911-023-02219-y

**Published:** 2023-09-15

**Authors:** Jixiao Wang, Chong Wang

**Affiliations:** 1 School of Physics, Mathematics and Computing, The University of Western Australia, Perth, 6009 Australia; 2 School of Business, Nanjing Audit University, Nanjing, 211815 China

**Keywords:** COVID-19, Erdos Renyl network, Prediction, Daily confirmed Cases, ICU bed, Mixed-effect model

## Abstract

The Chinese government relaxed the Zero-COVID policy on Dec 15, 2022, and reopened the border on Jan 8, 2023. Therefore, COVID prevention in China is facing new challenges. Though there are plenty of prior studies on COVID, none is regarding the predictions on daily confirmed cases, and medical resources needs after China reopens its borders. To fill this gap, this study innovates a combination of the Erdos Renyl network, modified computational model $SEIRS$, and python code instead of only mathematical formulas or computer simulations in the previous studies. The research background in this study is Shanghai, a representative city in China. Therefore, the results in this study also demonstrate the situation in other regions of China. According to the population distribution and migration characteristics, we divided Shanghai into six epidemic research areas. We built a COVID spread model of the Erodos Renyl network. And then, we use python code to simulate COVID spread based on modified $SEIRS$ model. The results demonstrate that the second and third waves will occur in July–September and Oct-Dec, respectively. At the peak of the epidemic in 2023, the daily confirmed cases will be 340,000, and the cumulative death will be about 31,500. Moreover, 74,000 hospital beds and 3,700 Intensive Care Unit (ICU) beds will be occupied in Shanghai. Therefore, Shanghai faces a shortage of medical resources. In this simulation, daily confirmed cases predictions significantly rely on transmission, migration, and waning immunity rate. The study builds a mixed-effect model to verify further the three parameters' effect on the new confirmed cases. The results demonstrate that migration and waning immunity rates are two significant parameters in COVID spread and daily confirmed cases. This study offers theoretical evidence for the government to prevent COVID after China opened its borders.

## Introduction

SARS-CoV-2, a novel coronavirus that causes COVID-19, was discovered at the end of 2019. The local spread began in China at the beginning of 2020. Then the virus spread rapidly around the world after April 2020. Due to the high mortality rate and lockdown, COVID has negatively impacted people’s health and the global economy. Consequently, scientists and researchers have done plenty of research regarding COVID, including nucleic acid reagents and vaccine developments. Also, some researchers concentrate on the daily confirmed case, and medical resource needs prediction. These researches provide a great deal of support when governments make decisions.

COVID is characterized by high variability. Hundreds of mutant strains and five majority strains were in the past three years, such as Alpha, Beta, Gamma, Delta, and Omicron. Up to now, the dominant strain is Omicron. The high transmission rate poses new challenges for epidemic prevention and control.

Before the Omicron spread, the original and Delta strain were the dominant strains. Due to the lower transmission rate of these two strains, the Chinese government relatively reached a balance between controlling the virus and economic development to some event. However, the current dominant strain is Omicron, and the transmission rate of Omicron is much higher than previous strains. The high transmission rate poses new challenges for epidemic prevention and control. Therefore, it becomes impossible to reach a balance. The Zero-COVID policy directly leads to economic recession in China [1, 2]. As an illustration, China GDP growth rate is only 0.3% in the second quarter of 2022 [3]. In addition, the mortality rate of Omicron is much lower than previous strains. The mortality rate of Omicron is 0.0093%. However, the original and Delta strains are 0.079% and 0.054%, respectively [4]. In general, Omicron is already a minimal hazard to the body, but the negative impact on the economy is enormous if the government maintains the Zero-COVID policy. Hence, relaxing the COVID policy has become necessary and urgent.

Fortunately, the Chinese government relaxed the Zero-COVID policy on 15 December and reopened the borders on 8 January. Nevertheless, China has a high population density, lacks medical resources, and residents have low antibodies against COVID. Millions of people may die if the government does not prepare and predict before opening the borders. Therefore, predicting medical resource needs and daily confirmed cases becomes more necessary. Although there are many prior studies on COVID, most concentrate on the original or Delta strain. The virus spreading in China is Omicron, which differs from the previous strains. However, no previous research predicts COVID spreading after China reopens the border. In this sense, the paper fills a gap. Since the demographics and characteristics of Shanghai are typical in China, it is an appropriate background to demonstrate the COVID situation in the whole of China.

This study merges Erdos Renyl networks, modified computational models $SEIRS$ and python code to predict daily confirmed cases and medical resources needs in Shanghai after reopening the borders. Firstly, we build Erdos Renyl networks in terms of Shanghai population density and migration characteristics. Secondly, according to the modified computational model $SEIRS$, we utilize python to simulate the epidemic spreading after determining the nine parameters. Finally, we obtain the results, likely daily confirmed cases, hospital bed needs, and ICU bed needs. Additionally, the study investigates which parameter of transmission, migration and waning immunity rate has the most significant impact on the COVID spreading. In this part, we calculate the overall coefficient by building a mixed-effect model. The highest overall coefficient represents the most significant parameter in COVID spreading.

The rest of the paper is structured as follows: Literature review section reviews the related literature. The progress of the simulation is in Methodology section. Mixed-effect model is presented in Impact of the parameters on daily confirmed cases section. Next, Discussion and Conclusion sections report the discussion and conclusion, respectively.

## Literature review

SARS-CoV-2, a novel coronavirus that causes COVID-19 emerged in China in late 2019 and was declared a pandemic by March 2020 [5, 6]. After that, COVID-19 spread throughout the world. The virus mutated into various strains over the past few years, causing different effects on human health. Due to the high variability of COVID cessing to analyse the severity of COVID, which is, there were hundreds of mutant strains in the past three years [7]. There are five main mutant strains since COVID-19 transmission. Having previously been defined as Alpha, Beta, Gamma, and Delta, Omicron became the fifth "variant of concern" by the World Health Organization in November 2021[8, 9]. Currently, Omicron is the dominant strain in the world. Researchers point out that these five main mutant strains differ entirely [10–12]. Under the studies, Omicron has more mutations than Alpha, Beta, Gamma, Delta, and wild-type, making it easier for the immune system to escape and speeding up the spread of the disease. However, Omicron’s hospitalization and mortality rate are significantly lower than previous.

So far, scholars worldwide have done a lot of research on COVID-19. They can be divided into several categories, likely daily confirmed cases prediction, sequelae analysis and reason of transmission.

Some researchers investigate the effect of temperature on virus transmission and conclude that higher temperature may sharply decrease the transmission speed [13, 14]. In the epidemic study, the most classic researches concentrate on predicting the daily confirmed cases and deaths. Numerous kinds of research are about these areas. Another study points out that the role of garbage in the transmission chain is more indirect in the sense that garbage has a complex relationship with public toilets [15]. Hence, pushing the ratio of public toilets to the local population in a city to its optimal level would help to reduce the total infection in a region.

Although various kinds of research are valuable, predicting the confirmed cases is always a typical research topic in infectious disease. Researchers use a mathematical model to investigate this problem. For example, [16, 17] are under ordinary differential equations (ODEs) to analyse the dynamics of local outbreaks of COVID. It predicted daily confirmed cases and the peak of the outbreak. The transmission rate is measured by varying the level of social distancing. Other papers also use another differential equation, such as the partial differential equation (PDE). The study utilizes PDE to predict the trend of COVID in Arizona, USA [18]. However, ODEs and PDE are pure mathematical models which lack empirical research evidence. Hence, the accuracy of results is usually much lower than others. However, some researchers use classic epidemic models. Some previous studies use the typical epidemic mathematical model $SIR$ and $SIRS$ to predict the COVID situation [19–21]. These prior studies investigate the number of susceptible, infected and recovered. However, some people may hospitalize or die during the COVID spreading. Obviously, the prior studies do not cover these areas.

As the classic epidemic mathematics models have some disadvantages, some researches explore new research areas. For instance, research utilises the self-created mathematical model to simulate the virus outbreak and how to control the epidemic [22]. It uses five parameters to build the model, such as the mortality rate for hospitalised people. In addition, prior study modifies the famous epidemic model $SIR$ [23]. In fact, COVID exhibits delay due to incubation periods and related phenomena. Hence, the study combines the basic model $SIR$ with delay differential equations (DDEs) and PDE. The above studies rely on differential equations and existing epidemic mathematical models. Using DDEs is advantageous for COVID prediction because it shows an incubation period that can improve models' accuracy. Additionally, DDEs and PDE mathematical models only need a little historical data, enhancing convenience. Nevertheless, these conventional models or equations influence prediction accuracy due to the various uncertainties. Therefore, attempting the computational techniques based on historical data may perform better.

Though some classifications or algorithms are not suitable for predicting daily confirmed cases, some prior studies attempt to utilise statistical models. They investigate how many confirmed cases are there in the future by exponential, non-linear, linear statistical models and Bayesian statistical models [24–26]. Firstly, the accuracy indicates that the performance of the Bayesian statistical model is worse than the statistical regression model. Thus, these studies are under the regression model. These papers predict the number of confirmed cases in Brazil, India and Myanmar. Although it achieves acceptable Mean Absolute Error (MAE) and Mean Squared Error (MSE), improving or optimising the models is difficult. Because the model always needs more independent variables for higher accuracy. Other studies combine the mathematical and statistical models, namely $SEIR$ mathematical model and the logistical statistical model. But the study mainly relies on a logistical statistical model to predict the trend of the COVID spread [27, 28]. It uses partial historical data to train and test the statistical model, which makes researchers comprehend the model's accuracy. The most significant reason is that the model lacks variables. That directly causes inaccuracy. On the whole, the regression model does not perform well. Consequently, the regression or statistical method is still not the most appropriate.

Some studies introduce how to use networks to simulate the virus spreading. Researchers describe that complex networks can utilize in infectious disease prediction, including star-shaped, power-law, and inhomogeneous W-graph [29–31]. The purpose of a complex network shows the relationship between vertex. Although these complex networks can show the relationship between vertex, which applies to virus prediction, the degree of each vertex is relatively fixed or impractical, influencing prediction accuracy. Another paper predicts the virus spreading based on social networks [32]. However, building a social network needs particular data, which is difficult for a significant population prediction. Hence, the social network is not suitable for this study. Previous study applies networks, computational language and programs to predict the trend of COVID [33]. It uses a 4-regular network to simulate the virus spreading. In other words, the simulation supposes the number of closed contacts of each person is four. However, this is different from reality. Fortunately, the Erdos Renyl network can modify the degree of each node easily, which can generally restore realistic scenarios. That is why this study applies the Erdos Renyl network.

Some research based on computational program language can simulate the COVID spreading arcuately. The research is the guideline for epidemic prediction [34]. It points out that a forecast that can be simulated in the most realistic environment is one of the most critical factors in COVID prediction. The computational programming simulation may be the best choice. Hence, this study also uses this method. Unfortunately, there is little research in this area.

These previous researches describe the various approaches studying COVID. The main results include the daily case prediction, factors influencing the morbidity and mortality of COVID and elements in the virus spreading. Although the results demonstrate the daily confirmed cases projection, most use statistical or mathematical models, which do not simulate COVID in an actual environment. Therefore, these studies still have room for improvement in prediction accuracy.

Currently, the dominant strain is B.A.7 [35]. However, many previous studies are based on the original strain, Delta or other strains. Since Omicron's transmission, hospitalisation, recovery, and mortality rate are very different from the previous strains, the prior studies do not reflect the current reality.

Moreover, resident COVID-19 antibody strength, population density, and government policy determine the virus's spread. The high population density is a distinctive feature of China, and Chinese residents do not have strong antibodies against COVID-19. The Chinese government implemented a strict epidemic prevention policy, Zero-COVID policy, until December 2022. Consequently, the COVID-19 prediction in other countries and China's COVID study based on data up to December 2022 is not indicative. There are no previous studies on COVID predictions for China reopening its borders or relaxing policies, which is urgent for the academic community to comprehend the COVID situation. Thus, this study focuses on BA.7 and the time after the Chinese government relaxed policies to predict COVID transmission, which is the novelty and uniqueness. Meanwhile, this study utilizes computational modelling to restore a realistic scene as possible and maximize the accuracy, rather than previous studies based on derivatives.

## Methodology

### Mathematical basis

#### ***Refined ***${\varvec{S}}{\varvec{E}}{\varvec{I}}{\varvec{R}}{\varvec{S}}$*** model***

In this study, we use a modified computational model $SEIRS$ to represent the epidemic spread, which means the susceptible individual may become infected. Then the infected person can recover from the virus. Finally, the individual will become susceptible again or die. Dead people will withdraw from the simulation. In order to recreate a scene as realistically as possible, model $SEIRS$ is like a computational model rather than ODE or PDEs model. According to the above explanation, the following expression or equation demonstrates the rule.

1 $$S\to E\to I\to R\to S$$

In Eq. (1), the first $S$ means susceptible individuals, $E$ is exposed person, $I$ represents infected people, $R$ indicates the people who recovered from the virus and these people cannot infect again. The second $S$ displays the recovered individuals who become susceptible again and people who died from the epidemic. After each simulation cycle, the situation of the second $S$ will be the beginning of the first $S$ in next simulation cycle. Since the sudden relaxation of covid policy, almost all residents are scared to be inflected. Due to the rapid infection of many people in a short time, most residents worked online, all campuses were closed, all restaurants only supported takeaway service, and all shopping centers strictly limited the pedestrian flow. Therefore, just a few numbers of the exposed population do not influence the covid situation significantly. That is why this study does not consider the exposed population. However, future studies or situations may include the exposed population. Therefore, this also adds an exposed population for future research.

In this paper, this study uses the python code ‘random’ to make the random parameter for the individual in each simulation and compare the random parameter with the set parameter. As an illustration, if the random migration rate is less than the set migration rate, then this individual will go to another group. However, if the random migration probability is larger or equal to the set migration rate, this individual does not move to another population group. According to the above explanation and Eq. (1), we refine it and obtain more details equations to demonstrate the epidemic spreading.

2 $$one a group\stackrel{\delta }{\to }person \left(node\right) another group$$

3 $$S\stackrel{\beta }{\to }I$$

4 $$I\stackrel{\mu }{\to }R$$

5 $$I\stackrel{\gamma }{\to }H\stackrel{\tau }{\to }R$$

6 $$I\stackrel{\gamma }{\to }H\stackrel{\rho }{\to }ICU\stackrel{\varphi }{\to }R$$

7 $$I\stackrel{\gamma }{\to }H\stackrel{\rho }{\to }ICU\stackrel{\sigma }{\to }D$$

8 $$R\stackrel{\varepsilon }{\to }S$$

Equation (2) means one node or (individual) goes to another population group based on migration rate ($\delta$). In reality, the system randomly chooses a node and decides whether go to another population group under the migration rate ($\delta$). If the person is susceptible, then it can be followed Eq. (3), which means the person may infect. In this case, the transmission rate ($\beta$) will apply.

Furthermore, the infected person has four outcomes. The first situation is like Eq. (4). This equation shows that the infected person may recover directly under the recovery rate ($\mu$). The second situation is like Eq. (5). According to hospitalization rate ($\gamma$) and hospitalization recovery rate ($\tau$), the person is hospitalized after infection and then recovers from the virus. The third one is that the infected person was hospitalized and admitted to the ICU for treatment. Afterwards, this individual also recovers. In this case, we utilize the hospitalization rate ($\gamma$), ICU hospitalization rate ($\rho$) and ICU hospitalization recovery rate ($\varphi$). This process is based on Eq. (6). The fourth situation is considerably different to the previous three. As an illustration, Although the infected patient underwent hospitalization and ICU treatment, he died. Equation (7) and mortality rate ($\sigma$) are applied in this case.

So far, Eqs. (4)-(7) are the situation in which the individual recovers. According to the $SEIRS$ rule, the recovered person may become susceptible again. Equation (8) illustrates that the recovered person becomes susceptible again because of the waning immunity rate ($\varepsilon$).

#### Total population

Due to the specificity of this study, which is mentioned in Refined SEIRS model section, only four parts in Eq. (1) are counted in the total population equation, namely $S$, $I$, $R$ and $S$. According to Refined SEIRS model section, $S$, $I$ and $R$ represent susceptible, infected, and recovered, respectively. The second $S$ means death people and the recovered person who may be infected again. However, only three states people exist at the beginning of the simulation. Consequently, the total population includes the number of susceptible, infected and, recovered, which is represented by $S\left(t\right)$, $I\left(t\right)$ and $R\left(t\right)$, respectively. The population means the total number of samples. Equation (2) demonstrates the whole population of simulation. ${N}_{population}$ implies the whole population of this simulation.

9 $${N}_{population}=S\left(t\right)+I\left(t\right)+R\left(t\right)$$

### Procedure of simulation

#### Erdos renyl network

At the beginning of the simulation, we use the python library ‘networkx’ to build the Erdos Renyl network to represent each individual and their closed contacts. Each node indicates an individual, and each individual has a different number of close contacts. In this model, the Erdos Renyl network is constructed based on two parameters, namely the number of nodes $N$ and the probability of each possible vertex connected with other nodes ${p}_{node}$. The degree distribution of Erdos Renyl network is a Binomial distribution.

11 $${p}_{k}={\left(\genfrac{}{}{0pt}{}{N-1}{k}\right)p}^{k}{(1-p)}^{N-1-K}$$

However, this study simulates the virus spreading in a large population community. Hence, the degree distribution is a Poisson distribution.

12 $${p}_{k}={e}^{-<k>}\frac{{<k>}^{k}}{k!}$$

Moreover, the property of Erdos Renyl network of this study is subcritical regime which means that graph is almost always disconnected with many components. Equation (13) shows this property.

13 $${p}_{node}<\frac{1}{N}$$

Furthermore, ${p}_{node}$ is followed by Eq. (14).

14 $${p}_{node}=\frac{average degree}{number of nodes (N)}$$

For instance, if the average degree is 4 and the population of network is 1000, then ${p}_{node}$ is 4e-3.

**Fig. 1:**
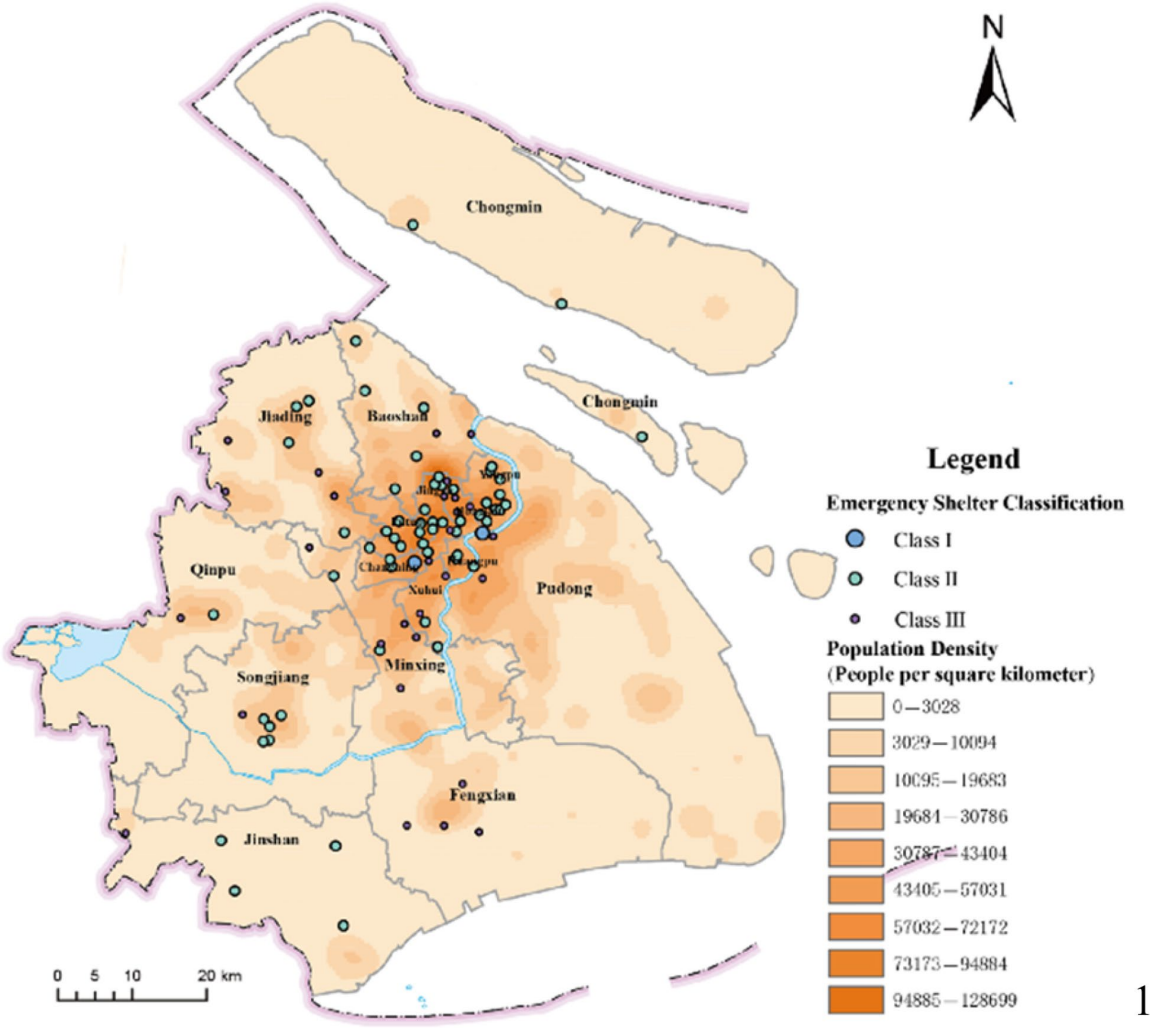
Shanghai population distribution and density (Fig. 1 originally from paper ‘A Multi-Indicator Evaluation Method for Spatial Distribution of Urban Emergency Shelters’. Permission obtained)

In this study, our research background is Shanghai, a city with a population of 25 million and 18 districts. As shown in Fig. 1, they show that the population density in the districts is quite different. For instance, the population density of all districts in the city centre is more than $20000 per/{km}^{2}$. Of these, Hongkou district population density is $32935 per/{km}^{2}$. In comparison, the density of the most rural district is between $500-2000 per/{km}^{2}$. Chongming district is only $539 per/{km}^{2}$. Thence, the population density in the city centre is significantly higher than in rural areas. It is obvious in Fig. 1 to see the differences in population density between districts.

**Fig. 2:**
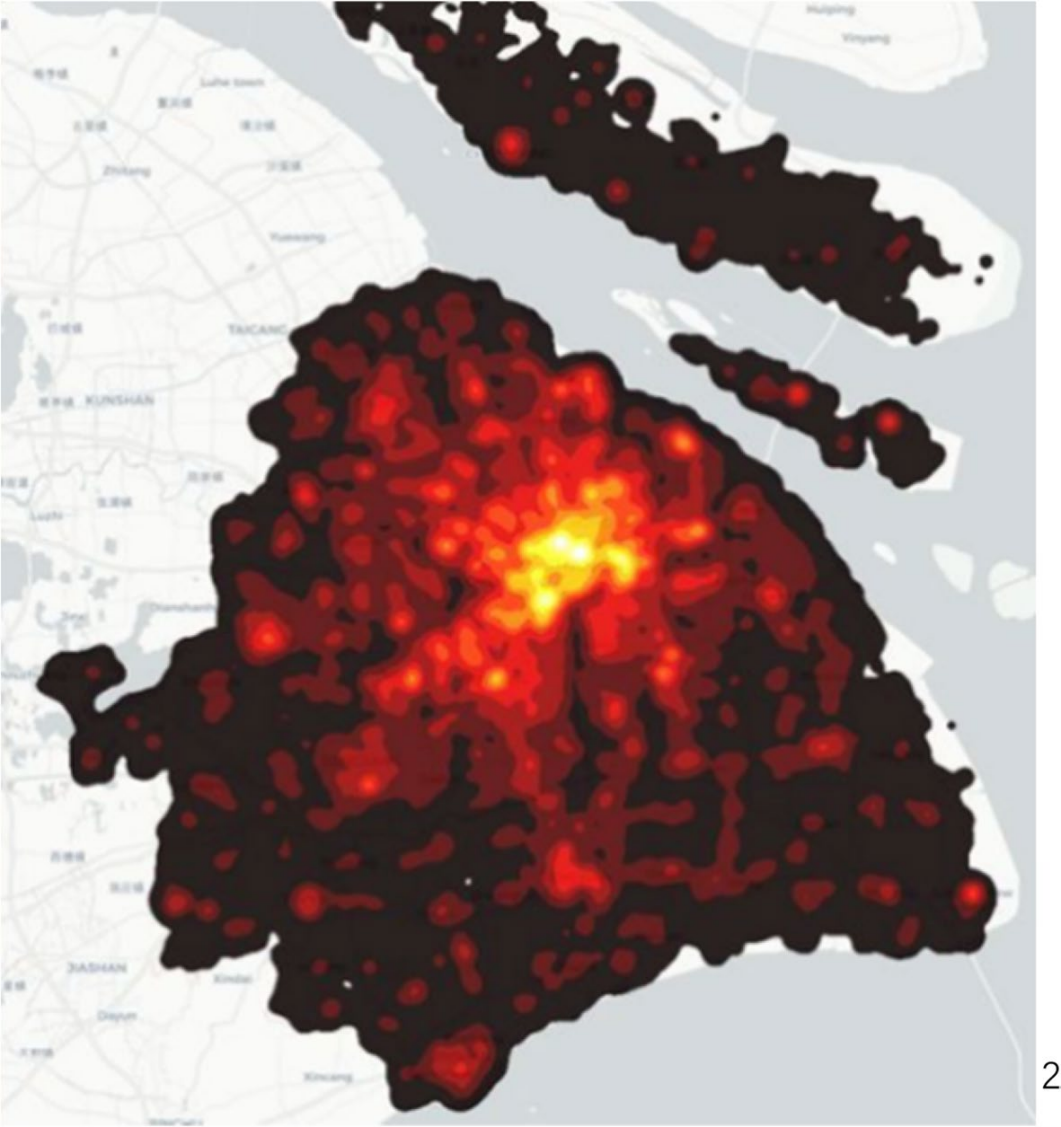
Population density at 10 a.m. (I acknowledge the image from https://www.sohu.com/a/235737015_691737, no copyright restrictions)

**Fig. 3:**
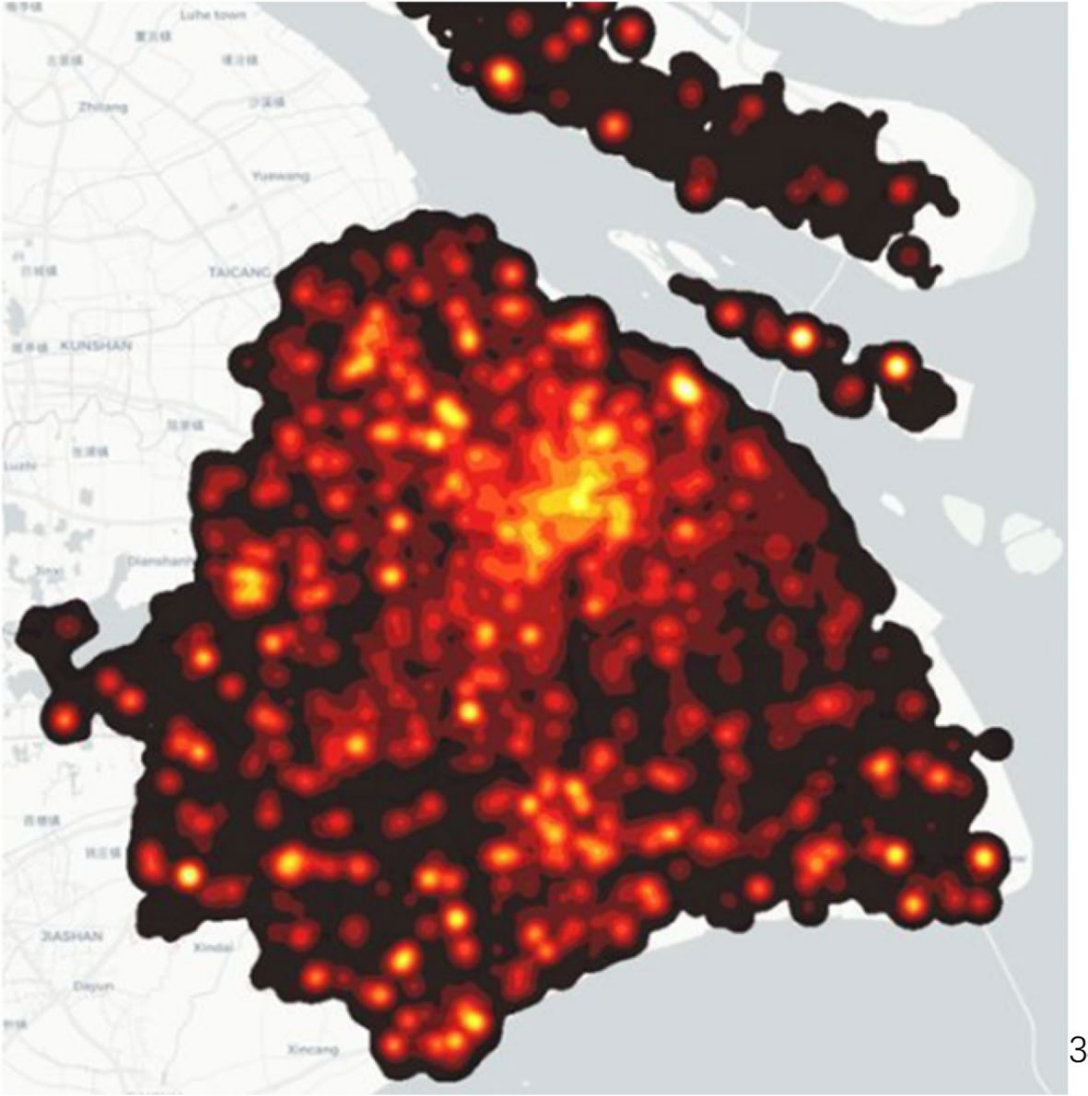
Population density at 10 p.m. (I acknowledge the image from https://www.sohu.com/a/235737015_691737, no copyright restrictions)

Figures 2 and 3 show the population density at 10 a.m. and 10 p.m., respectively. We found the most densely populated area is in the city centre at 10 a.m. However, people are in the rural area at 10 p.m. That is why lots of red dots are gathered in the city centre in Fig. 2 and scattered in rural areas in Fig. 3. These two figures demonstrate that people work in the city centre and reside in the rural area. In general, this describes the characteristic of population migration in Shanghai.

Under the population density in the districts, the characteristic of population migration, and virus transmission, we divide 18 districts into six epidemic research regions to analyse and predict the COVID-19 situation in Shanghai. At the same time, we use the python library ‘networkx’ to create six Erodos Renyl networks to represent six epidemic research regions based on population density and characteristics of migration [36, 37].

In this research, we create six epidemic research regions to predict and analyse the spread of COVID in Shanghai. For creating each network, we need to determine two parameters, namely the number of nodes $N$ and the probability of each possible vertex connected with other nodes ${p}_{node}$. $N$ implies the total population of the district represented by the network. To illustrate, the total population of Pudong district is 5.7 million, and Network 4 represents Pudong district. Thus, the number of nodes in Network 4 is 5.7 million. For obtaining ${p}_{node}$, the parameter is calculated by Eq. (13). In this study, the average degree is equal to the number of closed contacts. Since the total population of the districts $N$ has been determined, estimating the average degree of each network is the next step. Due to the discrepancy in population density of the districts, the number of closed contacts is also diverse. Accordingly, the number of average degrees is not the same in networks. For instance, Network 2 represents the highest population density district. So, the average degree of the network is also the highest. Detailed information on networks describes in Table 1.

**Table 1:** Detailed information of networks

No. network	Districts represented by the network	Number of nodes ($N$) in million	Average degrees	probability of each possible vertex connected with other nodes (${p}_{node}$)
1	Baoshan, Jiading and Qingpu	5.8	3	5.17 e-07
2	All city districts	6.8	7	1.03 e-06
3	Minhang and Fengxian	3.8	4	1.05 e-06
4	Pudong	5.7	5	8.77 e-07
5	Chongming	0.6	0.5	8.33 e-07
6	Songjiang and Jinshan	2.8	2	7.14 e-07

**Fig. 4:**
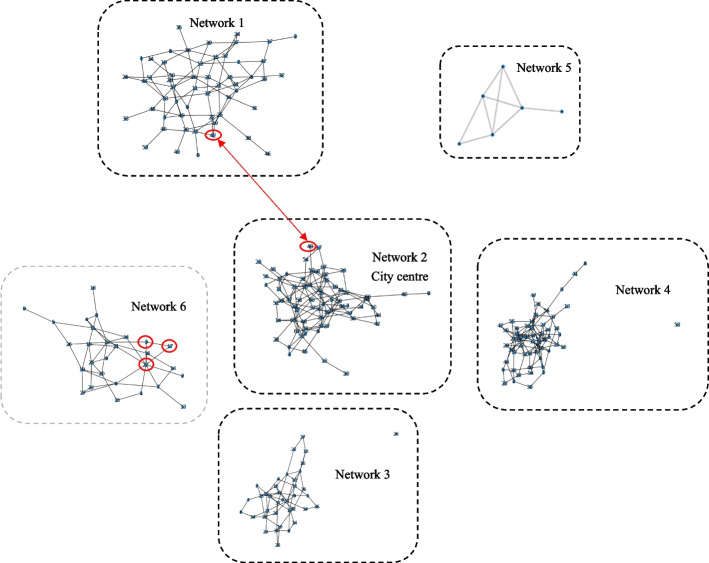
Schematic of Erodos Renyl network

It is difficult to show a full-scale network in this paper because each network has millions of nodes. Considering the readability, we shrink the population of the above six epidemic research regions 100,000 times to make six schematics of Erdos Renyl networks for demonstrating the epidemic spreading in Shanghai. For example, Network 1 has 5.4 million nodes. We only utilize 54 nodes in the schematics of Network 1. Figure 4 describes these six networks. In contrast, our simulation of the epidemic spreading in Shanghai is still based on the actual number of populations.

The numbers of degrees of each node vary in these six schematic Erodos Renyl networks, meaning each person has a different number of closed contacts. This is an advantage of the Erodos Renyl network because it reflects the reality. It is unlike other networks with constant degree of nodes, such as a regular network. Regular networks are “regular” because each node has the same number of links. In general, using the Erodos Renyl network has enormous advantages over the regular network or others. Using the Erodos Renyl network can simulate COVID spreading in a realistic environment.

#### Determining parameters

Although we build the simulation system in the previous part, we still need to determine and input all nine parameters, including migration rate ($\delta$), transmission rate ($\beta$) Etc. In this research, the parameters are provided by the Shanghai government. The first case was detected, which means the new wave began on 21 Nov 2022. From that date, the Shanghai government updates the number of new cases, hospitalisations and these nine parameters daily. Consequently, these figures vary every day. However, due to regulation, the complete data for the nine parameters used in this study cannot open to the public. Therefore, we only demonstrate the average for each parameter. They are shown in Table 2.

**Table 2:** Parameters used in the simulation

Parameter	Value	Parameter	Value
migration rate ($\delta$)	0.7	recovery rate ($\mu$)	0.998
transmission rate ($\beta$)	0.818	hospitalization recovery rate ($\tau$)	0.84
hospitalization rate ($\gamma$)	0.0052	ICU hospitalization recovery rate ($\varphi$)	0.32
ICU hospitalization rate ($\rho$)	0.00017	mortality rate ($\sigma$)	0.000093
waning immunity rate ($\varepsilon$)	0.00476		

#### Progress of simulation

The progress of the simulation is based on Erdos Renyl network, modified computational model $SEIRS$ and python code. Thence, the system uses above three techniques to describe the progress of simulation. Firstly, the system determines the initial infected. Although Shanghai experience a serious COVID wave and a long-time period of lockdown in the first half of 2022, Shanghai government maintain Zero-COVID policy, which has enabled Shanghai to maintain a consistently low growth in daily confirmed cases. From the end of September to 20 November 2022, the daily confirmed case is consistently at zero. However, one new case detected in Pudong district on 21 November 2022. This also marks the beginning of a new wave of epidemics. Therefore, the initial infected is one. As this initial infected was detected in Pudong district and Network 4 represents Pudong district, the system randomly identifies one node in Network 4 and update it is in infected status.

In this research, the system uses python code to make a probability for the individual in each process of simulation. Afterwards, the system compares the random parameter with the set parameters, such as transmission rate, recovery rate, waning immunity rate, Etc. As an illustration, if the random recovery probability is less than the set recovery rate, then this individual will recover. Also, the individual will be infected if the random recovery probability is larger or equal to the set recovery rate.

Throughout the simulation process, the first step is that the system randomly chooses a node to move to another population group which is based on migration rate ($\delta$) and Eq. 2. For example, as shown in Fig. 4, if the system randomly selects node 42 in Network 1 to go to another network, then the system will also randomly select a node from another network. For instance, the system chooses node 49 in Network 2 by simulation. 42 in Network 1 is infected, and node 49 in Network 2 is susceptible. So, after the migration, the original position of node 42 in Network 1 will be replaced by susceptible node 49 in Network 2. In contrast the original position of node 49 in Network 2 will be replaced by inflected node 42 in Network 1. This example is briefly marked in Fig. 4. Although we only introduce the migration between node 42 in Network 1 and node 49 in Network 2, each node can potentially migrate to other networks.

Afterwards, the system will check each susceptible node and its adjacent node in six networks. According to Fig. 4, if node 17 in Network 6 is susceptible, then the system will check whether its adjacent nodes are infected. This means the system will determine whether adjacent nodes 3 and 22 are infected. If its adjacent nodes are infected, and the random transmission rate is less than the transmission rate ($\beta$), indicating they meet Eq. (3), node 17 in Network 6 will become infected. This example is also briefly marked in Fig. 4.

Hereafter, the system focuses on infected individual. This process is based on recovery rate ($\mu$) and Eq. (4). If the node random recovery rate is less than the recovery rate ($\mu$), then the infected individual will become recovered. At the same time, the individual cannot be infected again if the person keeps recovered status.

Nevertheless, if the individual cannot recover, then the system tests whether the person will become hospitalized or still infected, which is based on hospitalization rate ($\gamma$) and Eq. (5). Additionally, the system also decides whether the hospitalized person still be hospitalized, ICU hospitalized or died. In detail, if the individual is hospitalized, then the simulation process updates the status of the individual is hospitalized or ICU hospitalized under the ICU hospitalization rate ($\rho$) and Eq. (6). In the same way, according to Eq. (7), if the individual’s status is ICU hospitalized and the random mortality rate is less than set mortality rate ($\sigma$), then the system labels the particular individual has died. Then, these dead people will withdraw from the simulation.

Although the infected person may become hospitalized, ICU hospitalized or die, they still have the opportunity to recover, except dead person. As an illustration, if the hospitalized person’s random ICU hospitalization rate is larger than the set ICU hospitalized rate ($\rho$) and the random recovery rate is less than the set hospitalization recovery rate ($\tau$), then this person is recovered. Suppose the random ICU hospitalization probability is larger than the ICU hospitalization rate ($\rho$), but the random hospitalization recovery rate is larger or equal to the set hospitalization recovery rate ($\varphi$). In that case, the individual is still hospitalized. Likewise, the ICU hospitalized status individual is similar to a hospitalized individual. The system concentrates on the ICU hospitalization rate ($\rho$) and ICU hospitalization recovery rate ($\varphi$).

**Fig. 5:**
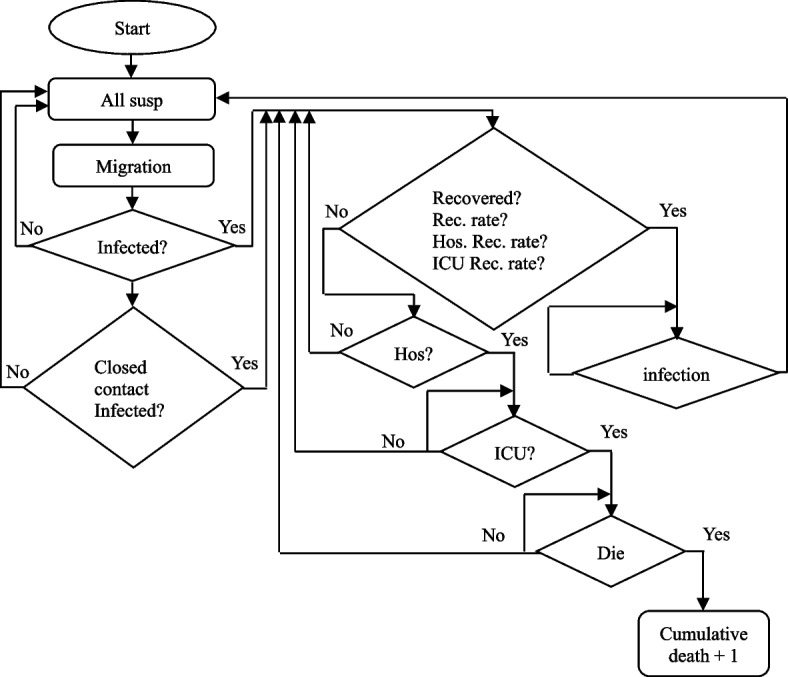
Simulation flow-process diagram

Finally, the system focuses on recovered individual. In this case, the waning immunity rate ($\varepsilon$) and Eq. (8) are two important indicators. The person will return to the susceptible state if the random waning immunity rate is less than the waning immunity rate ($\varepsilon$), the person will return to the susceptible state. Otherwise, it will keep recovering. Above explanations are the processes of the epidemic spread. It is also the simulation process for this system. Figure 5 shows the simulation process more intuitively.

### Prediction performance and results

So far, we have completed building the computational simulation. Hereafter, we input the nine parameters each day and run the simulation. Finally, we compare the simulation results with actual data to calculate the accuracy for verifying the simulation effect. The actual data includes the number of hospitalized, ICU hospitalized and death from 21 Nov 2022 to 31 Jan 2023. Daily confirmed cases from 21 Nov 2022 to 15 Dec 2022 are also included. Since the Shanghai government no longer collected daily confirmed cases on 15 Dec 2022, the actual daily case data ended by 15 Dec 2022.

After the simulation, we use Eq. (15) to calculate the accuracy. In Eq. (15), ${simulation}_{i}$ means the simulation results per day, ${actual}_{i}$ represents the actual data per day, and $n$ indicates the number of days predicted. Lastly, accuracy is derived by calculating the cumulative deviation.

15 $$1-\frac{\sum_{i=1}^{n}\left|{simulation}_{i}-{actual}_{i}\right|}{n}$$

**Fig. 6:**
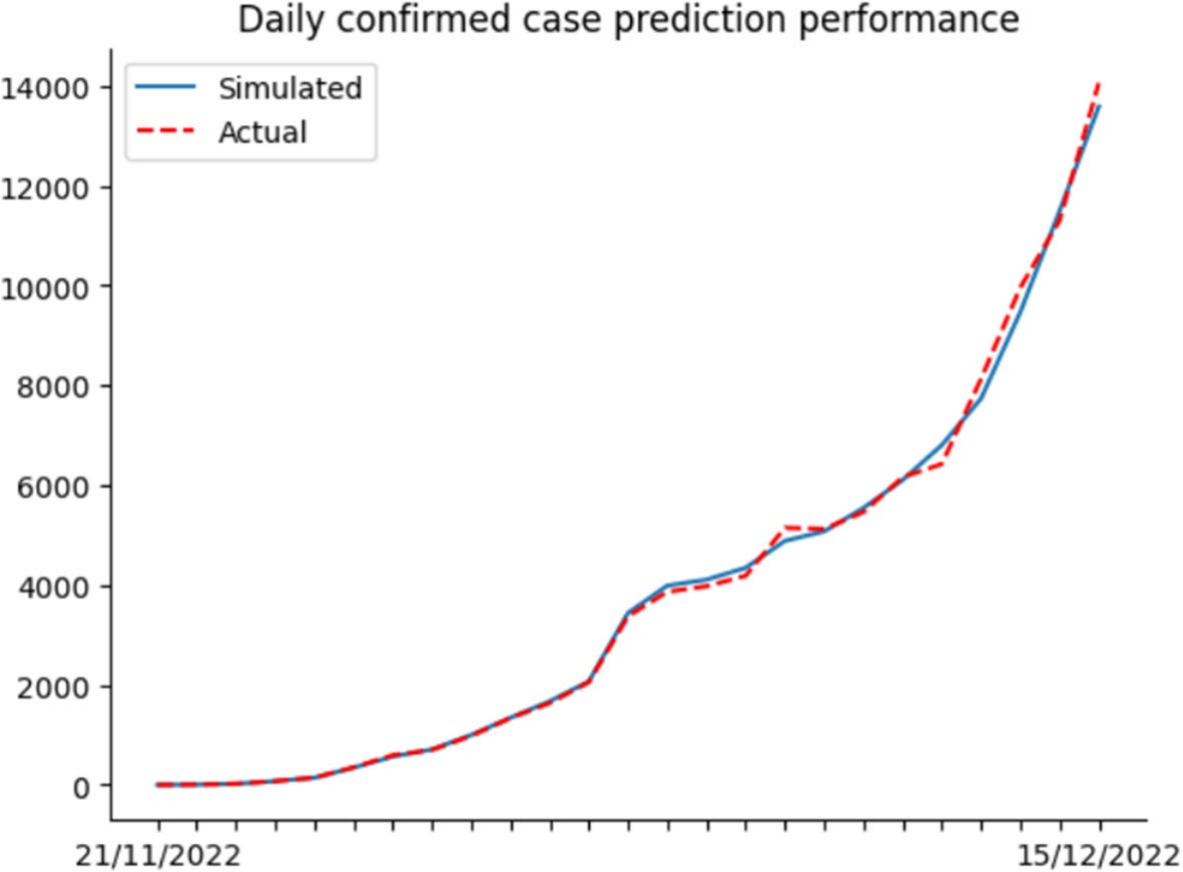
Dily confirmed case prediction performance

**Fig. 7:**
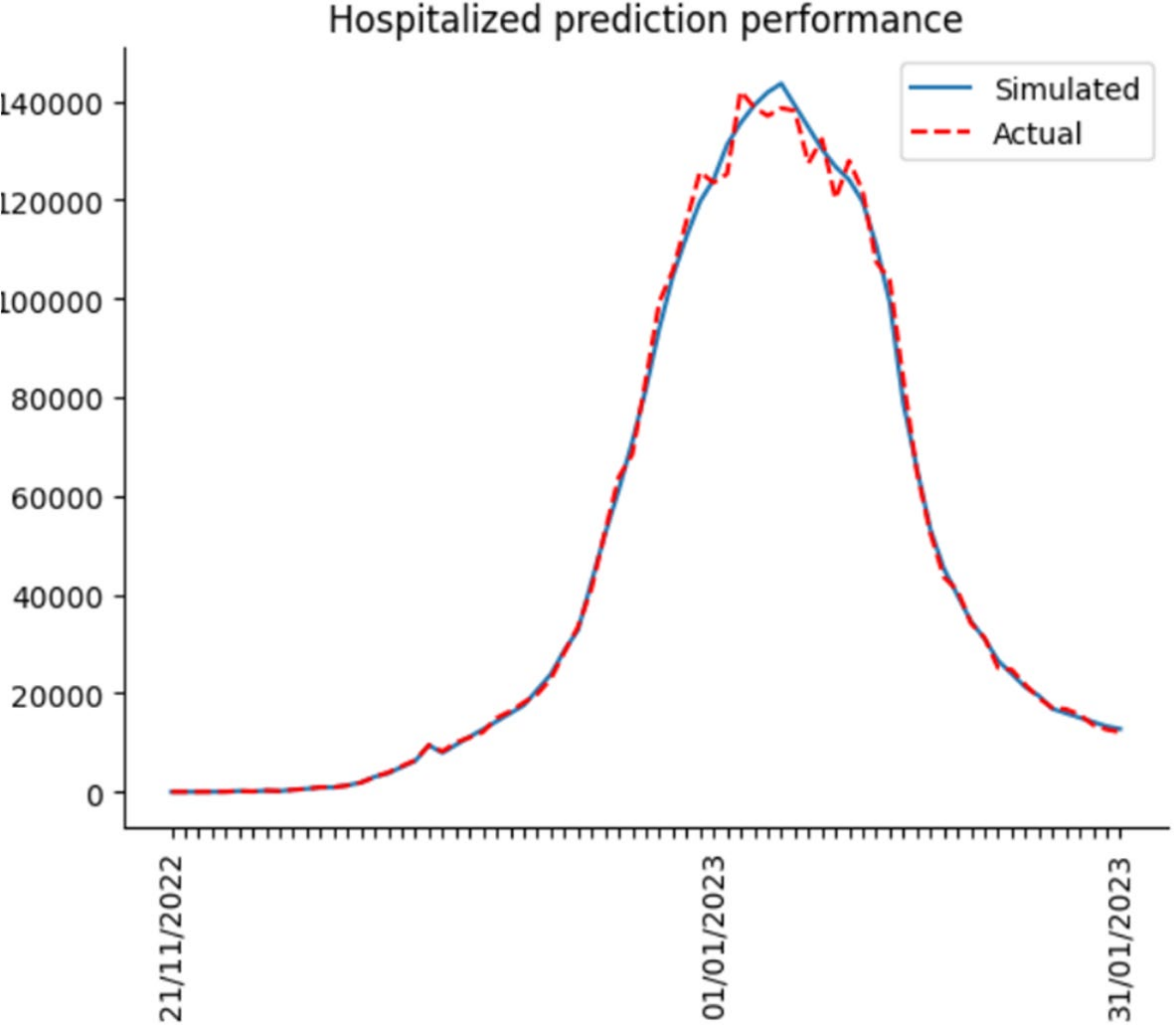
Hospital bed needs prediction performance

**Fig. 8:**
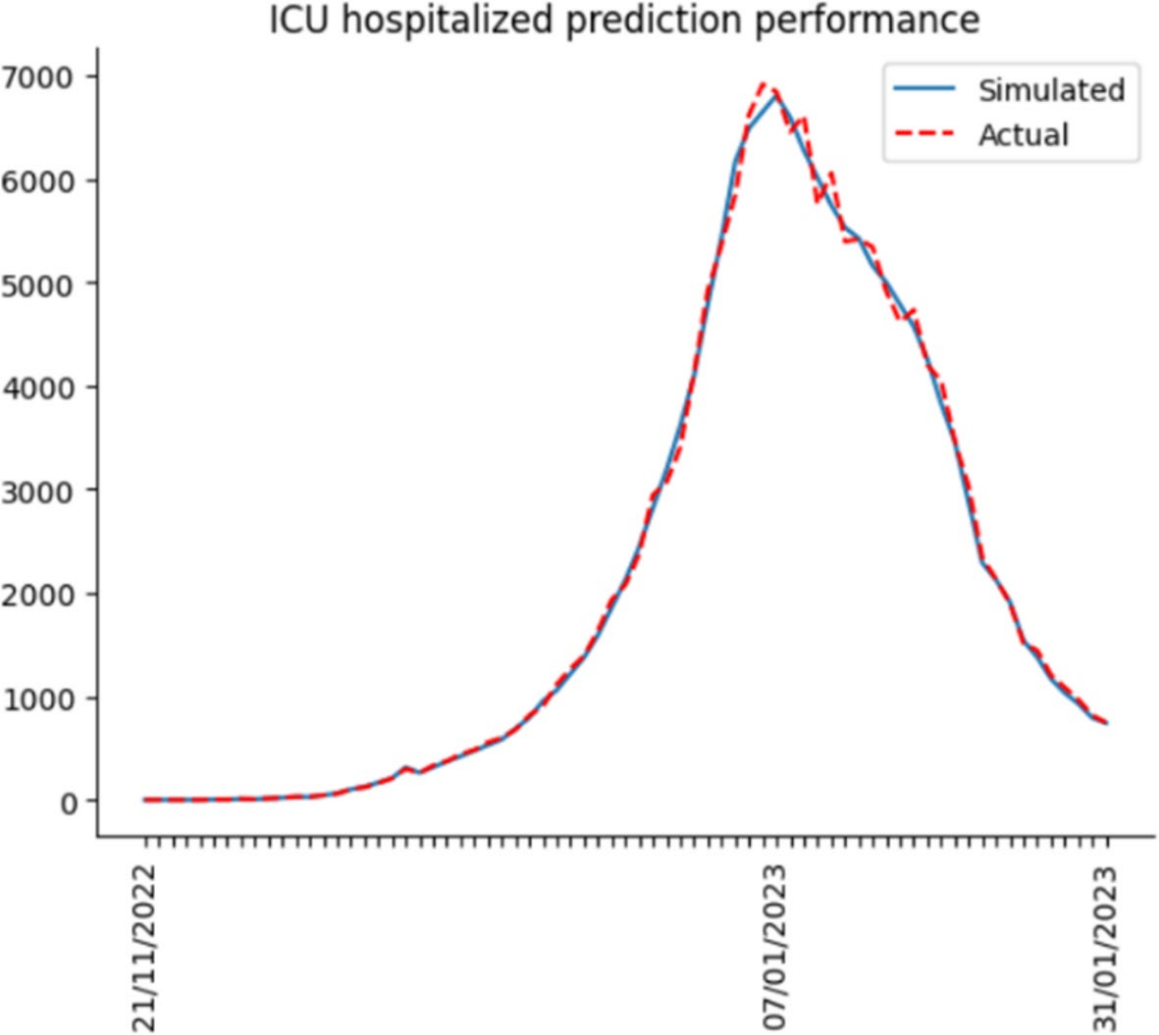
ICU bed needs prediction performance

**Fig. 9:**
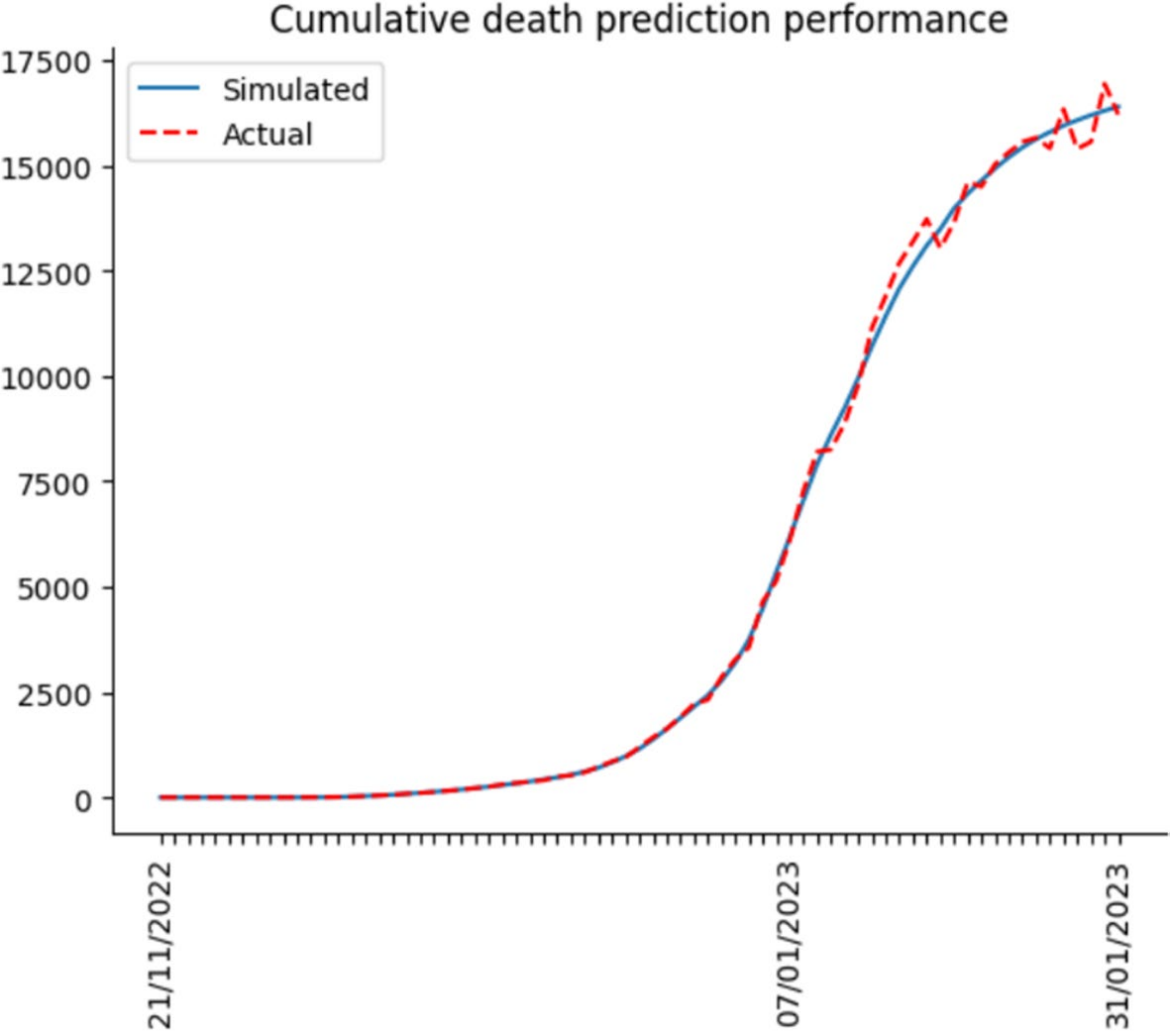
Cumulative death prediction performance

Figures 6, 7, 8 and 9 illustrate the deviation between actual and simulated data in the daily case and medical resource needs. The prediction accuracies of daily confirmed cases, hospital bed needs, ICU bed needs, and cumulative deaths are 0.954, 0.962, 0.951 and 0.968, respectively. And the overall accuracy of the simulation is 0.959. In general, the simulation performs quite well, and the result is reliable.

Since we demonstrate the accuracy of the simulation is outstanding, we use it to forecast the daily confirmed cases, hospital bed needs, ICU bed needs and death in the following year. In addition, we use the historical data from the Shanghai government as parameters used in the simulation [38] point out that although omicron is highly mutable, there has never been a strain in the last six months that was very different from the previous strains. In other words, there is no significant change in the rate of transmission or pathogenicity of the virus. Therefore, the historical data of nine parameters still applies to future outbreak projections. Through the projections, we make the following figures to reveal the COVID trend in Shanghai after China reopening its borders.

**Fig. 10:**
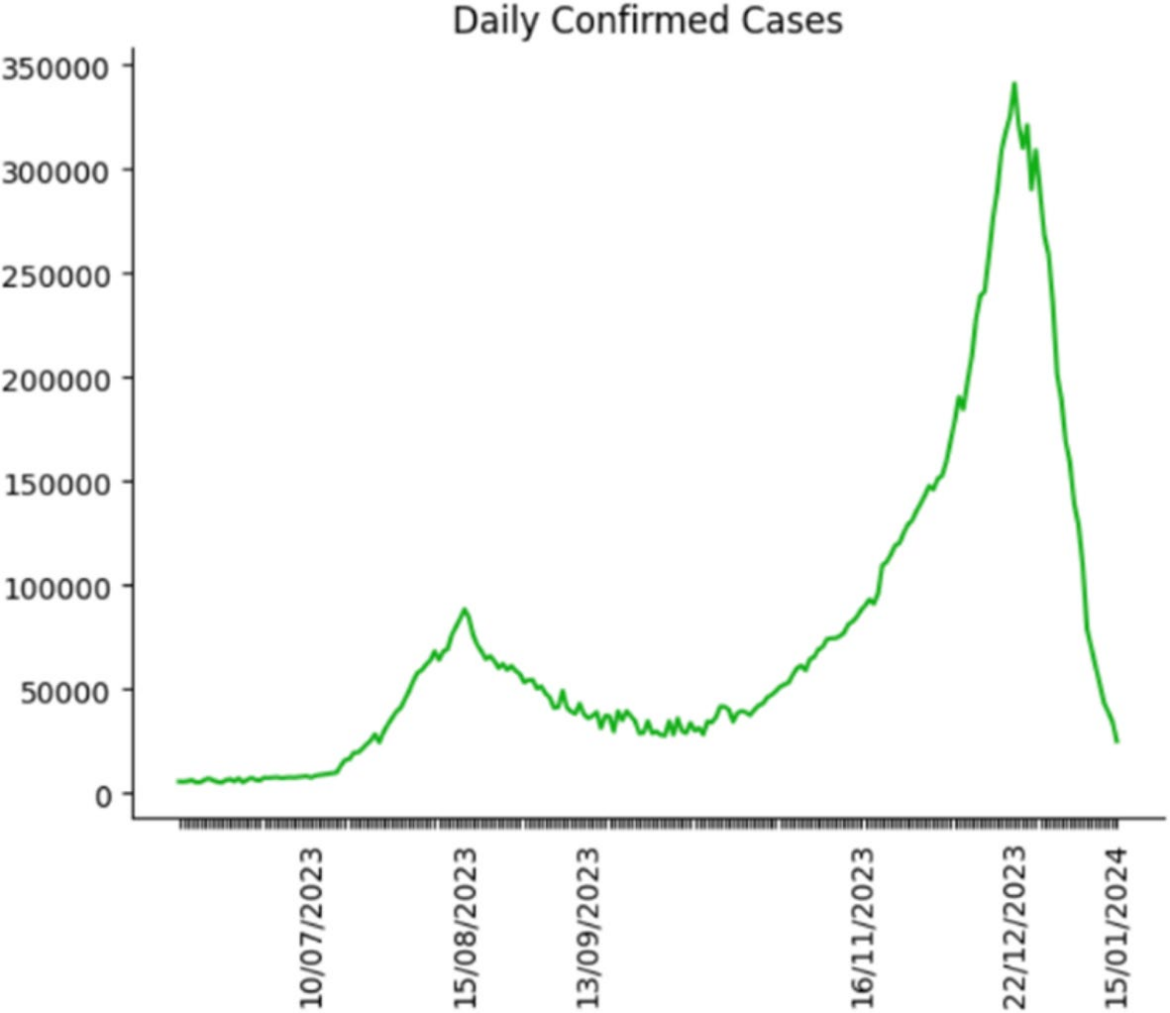
Daily confirmed cases prediction

**Fig. 11:**
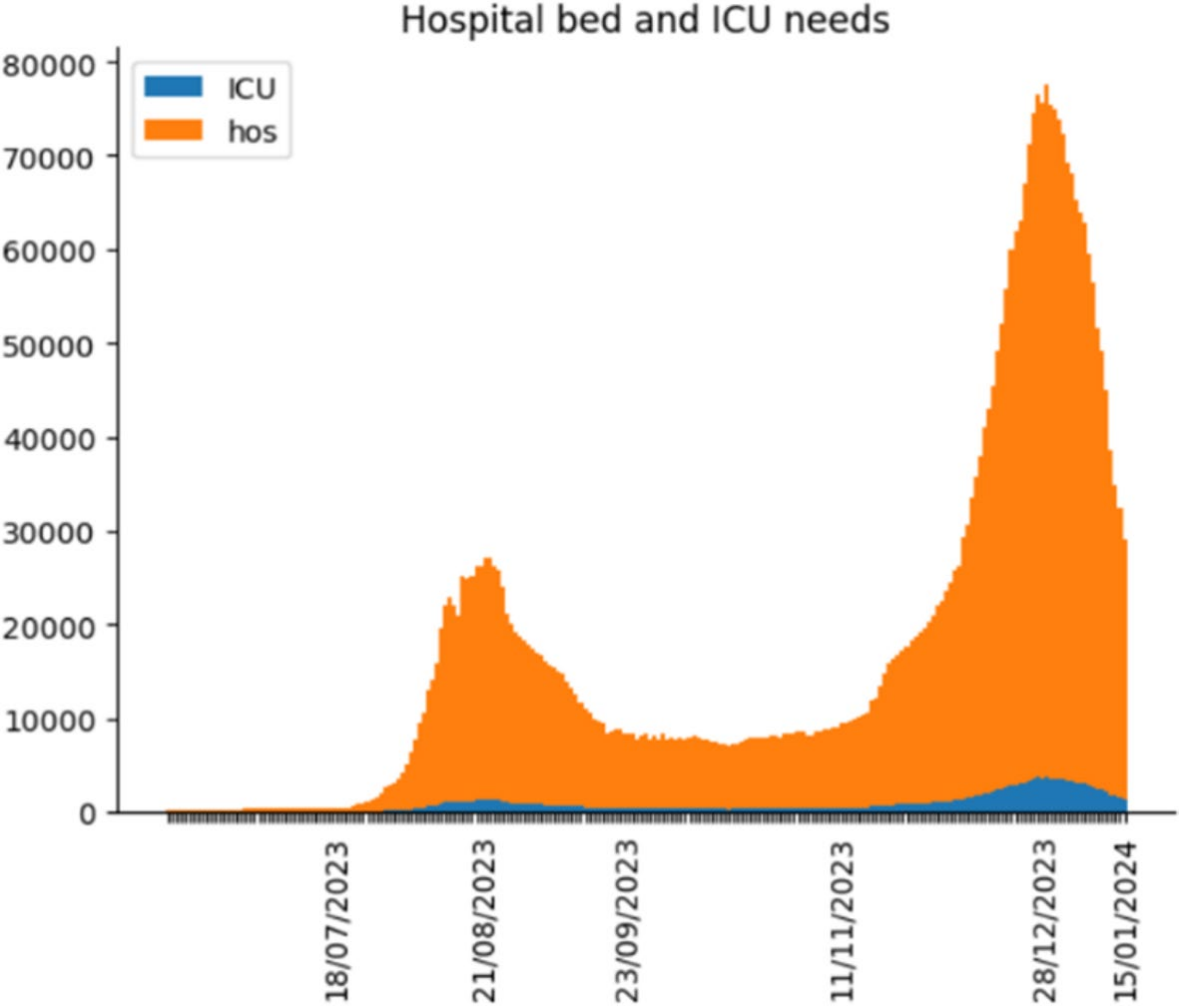
Hospital and ICU bed needs prediction

**Fig. 12:**
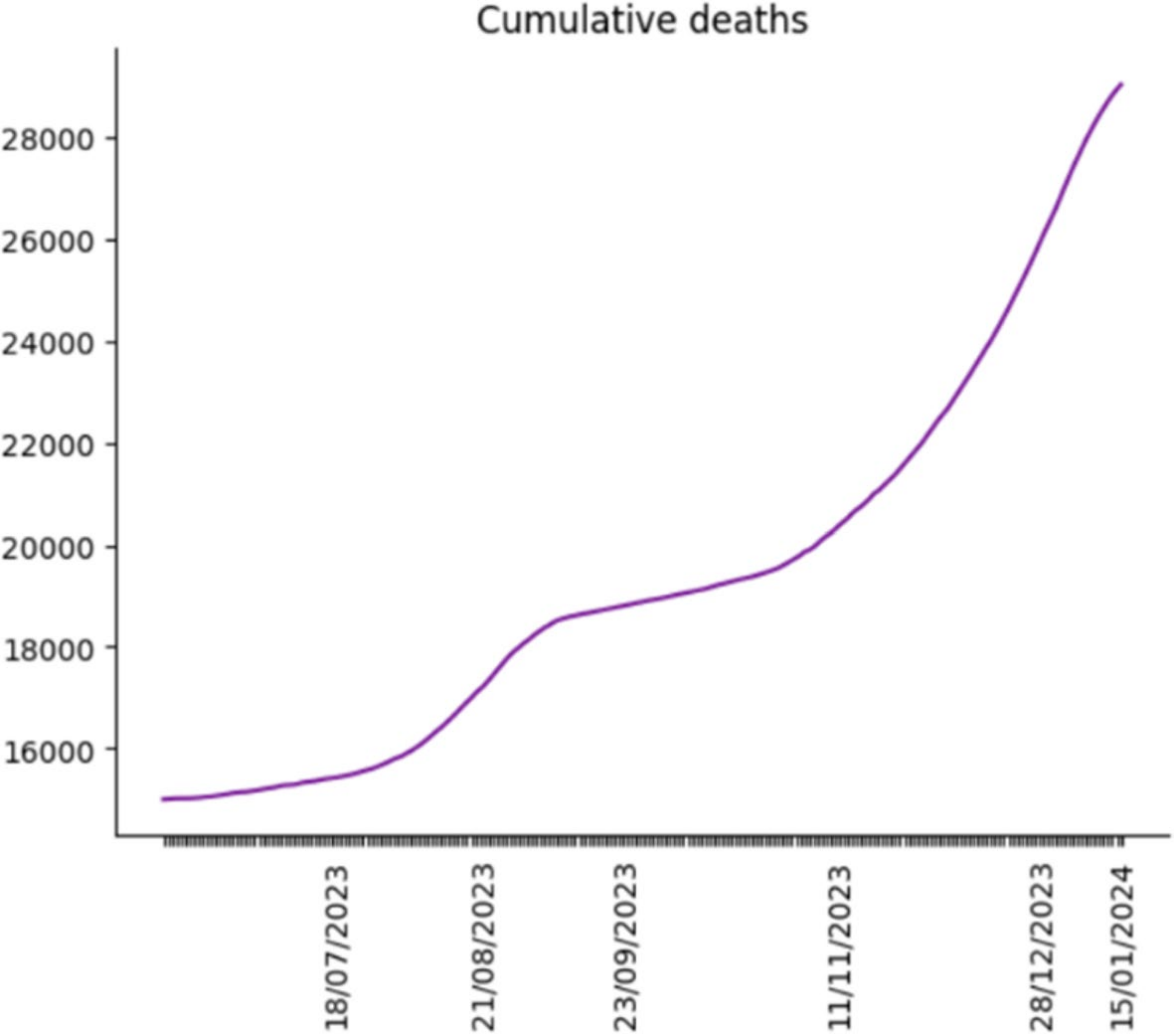
Cumulative deaths prediction

Figures 10, 11 and 12 demonstrate the COVID trend prediction in Shanghai after China reopening the borders, including daily confirmed cases, hospital bed needs, ICU bed needs and cumulative deaths. Due to the readability, figures only label the critical date in the x-axis, such as the peak, start and end date of each wave. Figures 10, 11 and 12 respectively illustrate the daily confirmed cases, hospital bed needs, ICU bed needs, and cumulative deaths in Shanghai after China reopening the borders. It is found in Figs. 10, 11 and 12, although the first wave of the outbreak is over, the second and third COVID waves will outbreak in the future. Therefore, the government and residents should still attach importance to COVID prevention.

## Impact of the parameters on daily confirmed cases

### Building and optimizing statistical model

Through the previous predictions, the results demonstrate that Shanghai will experience the second and third waves in 2023. If COVID leads to too many people being infected, it hurts the economy deeply [39]. Thence, it is necessary for the government to minimise the number of daily confirmed cases in each wave of the epidemic as few as possible. Therefore, identifying the important factors that can influence the number of daily confirmed cases is the crucial section. This can make the government provide more scientific guidance to citizens for self-prevention, which decreases the negative impact on the economy and protect people’s health.

In the simulation, three parameters can influence the daily case: transmission rate, migration rate and waning immunity rate. Thus, we use historical data from the Shanghai Government relating to these three parameters and the parameter ‘recovery rate’ to build the statistical model. We need to add the variable ‘recovery rate’ in the statistical model because adding the variable ‘recovery rate’ makes the simulation complete and more realistic. Since hospitalized and dead people only count a few portions of the whole population, it does not affect the result of the statistical model. Thus, the statistical model does not include the variables for hospitalization death, such as variable ‘hospitalization rate’, ‘mortality rate’ and so forth. In general, the dependent variable is the daily confirmed case. The independent variables include transmission rate, migration rate, waning immunity rate and recovery rate. After building and optimizing the statistical model, we calculate the overall coefficient of each parameter. The highest overall coefficient means the maximum influence of daily confirmed cases.

#### Linear and multiple polynomial model

Our first step utilizes multiple linear regression with homogeneous variance. The classical linear model (LM):

$$Y=X\beta +\varepsilon , \varepsilon \sim N(0, {\sigma }^{2}I)$$

Implies that outcomes ${Y}_{i}$ are independent and normally distributed:

$${Y}_{i} \sim N\left({\mu }_{i}{, \sigma }^{2}\right), i=1,\dots ,n$$

where ${\mu }_{i}={X}_{i}^{T}\beta$. Note that all ${Y}_{i}$ have the same variance, namely ${\sigma }^{2}$. Hence, the simple linear regression model is

16 $${case}_{i}={\beta }_{0}+{\beta }_{1}infectiousRate+ {\beta }_{2} recoveredRate+ {\beta }_{3} immuniteRate+ {\beta }_{4} immigrateRate+ {\varepsilon }_{i}$$

Then we use R code ‘lm’ to make a LM. However, we found the residual is too large and we notice each variable residual plot has quadratic pattern. Therefore, we use quadratic, cubic and other powers to increase the fitted model accuracy until hypothesis test’s p-value is greater than 0.05 which means we accept null hypothesis. Finally, the best fitted model has shown in below:

17 $${case}_{i}={\beta }_{0}+ {\beta }_{1} infectiousRate+{\beta }_{2} {infectiousRate}^{2}+ {\beta }_{3 }{infectiousRate}^{3}+ {\beta }_{4 }{infectiousRate}^{4}+ {\beta }_{5} recoveredRate+ {{\beta }_{6 }recoveredRate}^{2}+{{\beta }_{7 }recoveredRate}^{3}+{\beta }_{8} immunityRate+{{\beta }_{9 }immunityRate}^{2}+ {{\beta }_{10 }immunityRate}^{3}+{\beta }_{11}immigrateRate+ {{\beta }_{12} immigrateRate}^{2}+ {{\beta }_{12} immigrateRate}^{3}+{\varepsilon }_{i}$$

#### Mixed-effect model

The above machine learning algorithm (linear or multiple regression) apply constant variance $var\left[{Y}_{i}\right]={\sigma }^{2}$. However, the heterogeneous variances are more appropriate in reality, rather than homogeneous variance (constant variance). We now relax the constant variance assumption and assumption that $var\left[{Y}_{i}\right]={\sigma }_{i}^{2}$. Therefore, a LM with heterogeneous variance can be formulated as:

$${Y}_{i}= {x}_{i}^{T}\beta +{\varepsilon }_{i}, i=1,\dots ,n$$

with ${\varepsilon }_{i}\sim N(0, {\sigma }_{i}^{2})$ and the ${\varepsilon }_{i}$ are independent. Or, in matrix notation,

$$Y= X\beta +\varepsilon , \varepsilon \sim N(0,{\sigma }^{2}R)$$

where R is a diagonal matrix.

The simplest way to introduce heteroscedasticity and, at the same time, to reduce the number of variance parameters, is to assume that the variance of ${\varepsilon }_{i}$ is equal to a known proportion of one (unknow) parameter ${\sigma }^{2}$. More specifically, we may associate with every observation $i$ a known constant ${w}_{i}>0$ and assume that

$$var\left[{\varepsilon }_{i}\right]=var\left[{Y}_{i}\right]=\frac{{\sigma }^{2}}{{w}_{i}}$$

For instance, if ${Y}_{i}$ is the average of ${n}_{i}$ observations (all with the same covariates) and the original observations were homogeneous. We can consider the transformed model:

$${w}_{i}^{1/2}{y}_{i}={\beta }_{0}{w}_{i}^{1/2}1+{\beta }_{1}{w}_{i}^{1/2}{x}_{i1}+\dots +{\beta }_{p}{w}_{i}^\frac{1}{2}{x}_{ip}+{w}_{i}^\frac{1}{2}{\varepsilon }_{i}, i=1,\dots , n$$

Then $var\left[{w}_{i}^\frac{1}{2}{\varepsilon }_{i}\right]={\sigma }^{2}$--- we are back at a homogeneous LM. This motivates estimates / estimators of $\beta$ via a weighted sum of squares:

$$\sum_{i=1}^{m}{w}_{i}{({y}_{i}-{x}_{i}^{T}\beta )}^{2}={(y-X\beta )}^{T}W(y-X\beta )$$

where$W=diag({w}_{1},{w}_{2},\dots , {w}_{n}$) is a diagonal matrix which leads to

$${\widehat{\beta }}_{WLS}={({X}^{T}WX)}^{-1}{X}^{T}{W}_{y}$$

$${\widehat{\sigma }}_{WLS}^{2}=\frac{1}{n-p-1}{(y-X{\widehat{\beta }}_{WLS})}^{T}W(y-X{\widehat{\beta }}_{WLS})$$

A more general and flexible way to introduce variance heterogeneity is by means of a variance function g(.). The variance of the residual errors $var\left[{\varepsilon }_{i}\right]$, is expressed as follows:

$$var\left[{\varepsilon }_{i}\right]={\sigma }^{2}{\mathrm{g}}^{2}(\delta ,{ \mu }_{i}; {v}_{i})$$

where ${\mu }_{i}=E\left[{Y}_{i}\right]={x}_{i}^{T}\beta$, $\sigma$ is a scale parameter, ${v}_{i}$ is a vector of (known) covariates defining the variance function for observation $i$, while the vector $\delta$ contains a small set of variance parameters, common to all observations. Note that, because function g(.) involves ${\mu }_{i}$, it in fact depends on $\beta$, too. It is worth underscoring here that the parameter ${\sigma }^{2}$ in general should be interpreted as a scale parameter. This is the classical LM with homogeneous variance in which ${\sigma }^{2}$ can be interpreted as residual error standard deviation. Note that, g(.) should, strictly speaking, be referred to as a function modelling standard deviation, not variance. However, the term variance function is commonly used when referring to g(.).

In this study, we use two variance functions, namely different variances per stratum (varIdent) and power of a covariate (varPower).

For varIdent, this class represents a variance model with different variances for each level of a stratification variable s, taking values in the set {1,2, …, S},

$$var\left[{\varepsilon }_{i}\right]={\sigma }^{2}{\delta }_{{s}_{i}}^{2 } \mathrm{corresponding} \mathrm{to} \mathrm{g}\left({s}_{i, } \delta \right)={\delta }_{si}$$

This variance model uses $S+1$ parameters to represent $S$ variances and, therefore, is not identifiable. To achieve identifiability, some restriction needs to be imposed on the variance parameters $\delta$. ${\delta }_{i}=1$ is used, so that ${\delta }_{I}$, $I=2,\dots , S$, represent to ratio between the standard deviations of the /th stratum and the first stratum. By definition, ${\delta }_{I}>0$, $I=2,\dots , S$.

For varPower, the variance model represented by this class is

$$var\left[{\varepsilon }_{i}\right]={\sigma }^{2}{\left|{v}_{i}\right|}^{2\delta } \mathrm{corresponding to} \mathrm{g}\left({s}_{i}, \delta \right)={\left|{v}_{i}\right|}^{\delta }$$

These main arguments to varPower are value and form, which specify, respectively, an initial value for $\delta$, when this is allowed to vary in the optimization, and a one-sided formula with the variance covariate. Note that, when ${v}_{i}=0$ and $\delta >0$, the variance function is 0 and the variance weight is undefined. Therefore, this class of variance functions should not be used with variance covariates that may assume the value 0.

Afterward, we use R code ‘glm’ to build the mixed-effect model based on the Eq. 16. We apply varIdent and varPower variance function. Thus, we get the two mixed-effect statistical model. The first model with varIdent represents as follow.

18 $$\begin{array}{c}{case}_{it}= {\beta }_{0t}+{\beta }_{1}{infectiousRate}_{i}+{\beta }_{2t}{infectiousRate}_{i}^{2}+{\beta }_{3t}{infectiousRate}_{i}^{3}+{\beta }_{4t}{infectiousRate}_{i}^{4}+{\beta }_{5t}{recoveredRate}_{i}+{\beta }_{6t}{recoveredRate}_{i}^{2}+{\beta }_{7t}{recoveredRate}_{i}^{3}+ {\beta }_{8t}{immunityRate}_{i}+{\beta }_{9t}{immunityRate}_{i}^{2}+{\beta }_{10t}{immunityRate}_{i}^{3}+ {\beta }_{11t}{immigrateRate}_{i}+{\beta }_{12t}{immigrateRate}_{i}^{2}+{\beta }_{13t}{immigrateRate}_{i}^{3}+ {\varepsilon }_{it}\\ {\sigma }_{it}=\sigma {\mathrm{g}}_{it}=\sigma \mathrm{g}(\left({\delta }_{1, } {\delta }_{2, }{\delta }_{3, }{\delta }_{4}\right);{day}_{it}\\ =\left\{\begin{array}{c}\sigma ({{day}_{it})}^{{\delta }_{1}} for infectious rate\\ \sigma ({{day}_{it})}^{{\delta }_{2}} for recovered rate\\ \sigma ({{day}_{it})}^{{\delta }_{3}} for immunity rate\\ \sigma ({{day}_{it})}^{4} for immigrate rate\end{array}\right.\end{array}$$

The first model with varPower illustrates as follow.

19 $$\begin{array}{c}{case}_{it}={\beta }_{0t}+{\beta }_{1}{infectiousRate}_{i}+{\beta }_{2t}{infectiousRate}_{i}^{2}+{\beta }_{3t}{infectiousRate}_{i}^{3}+{\beta }_{4t}{infectiousRate}_{i}^{4}+ {\beta }_{5t}{recoveredRate}_{i}+{\beta }_{6t}{recoveredRate}_{i}^{2}+{\beta }_{7t}{recoveredRate}_{i}^{3}+ {\beta }_{8t}{immunityRate}_{i}+{\beta }_{9t}{immunityRate}_{i}^{2}+{\beta }_{10t}{immunityRate}_{i}^{3}+ {\beta }_{11t}{immigrateRate}_{i}+{\beta }_{12t}{immigrateRate}_{i}^{2}+{\beta }_{13t}{immigrateRate}_{i}^{3}+ {\varepsilon }_{it}\\ {\sigma }_{it}=\sigma {\mathrm{g}}_{it}=\sigma \mathrm{g}\left(\delta ,{\mu }_{it}\right)=\sigma {({\mu }_{it})}^{\delta }\end{array}$$

#### Multiple polynomial model vs mixed-effect model

So far, we have built three statistical models: one multiple-polynomial model and two mixed-effect models. Therefore, our next step is determining whether the mixed-effect model is better than multiple polynomial model.

Firstly, multiple polynomial model is better or the mixed-effect model with varIdent. R code ‘ANOVA’ is be used in this test. This command tests:

$${H}_{0}: {\sigma }_{1}^{2}={\sigma }_{2}^{2}={\sigma }_{3}^{2}={\sigma }_{4}^{2} \mathrm{VS }{H}_{1}: {at least two \sigma }_{t}^{2} differ$$

Test statistic (likelihood-ratio test) has asymptotically a ${X}_{3}^{2}$ distribution, i.e., a ${X}^{2}$ distribution with 3 degrees of freedom. Also, the *p*-value is < 0.001. Hence, we believe that alternative hypothesis (${H}_{1}$) is our preferred. In other words, mixed-effect model is a more appropriate model. Secondly, we still use R code ‘ANOVA’ to test whether varIdent is better than model with varPower. This command tests:

$${H}_{0}: {\delta }_{1}={\delta }_{2} \mathrm{VS }{H}_{1}: {\delta }_{1}\ne {\delta }_{2}$$

Test statistic (likelihood-ratio test) has asymptotically a ${X}_{1}^{2}$ distribution, i.e., a ${X}^{2}$ distribution with 1 degree of freedom. Additionally, the p-value is < 0.001. Hence, we believe that alternative hypothesis (${H}_{1}$) is our preferred. In other words, mixed-effect model with varPower is the best model. We also use Akaike information criterion (AIC) to test the performance of the statistical models. Table 3 represents the details of AIC test result and degree of freedom (DF).

**Table 3:** Details of AIC test result

Statistical model	DF	AIC
Multiple-polynomial model	6	4016.19
Mixed-effect model with varIdent	10	2559.81
Mixed-effect model with varPower	11	1934.23

According to the above result, the Mixed-effect model with varPower is the best statistical model because of minimum AIC and relatively lower DF.

#### Result of statistical model

After the analysis, ANOVA test points out the mixed-effect model with varPower is the most appropriate statistical model. The coefficient of final model shows in Table 4.

**Table 4:** Coefficient of mixed-effect model

Variable	Coefficient	*P*-value	Variable	Coefficient	*P*-value
intercept	917.15	2e-16	${recovRate}^{3}$	-209.69	2e-16
$infecRate$	290.441	2e-16	$immuniRate$	716.049	2e-16
${infecRate}^{2}$	1877.73	2e-16	${immuniRate}^{2}$	582.49	2e-16
${infecRate}^{3}$	3495.39	2e-16	${immuniRate}^{3}$	-140.03	2e-16
${infecRate}^{4}$	-1612.44	2e-16	$immigraRate$	735.614	2e-16
$recovRate$	-910.72	2e-16	${immigraRate}^{2}$	616.65	2e-16
${recovRate}^{2}$	1113.47	2e-16	${immigraRate}^{3}$	-157.74	2e-16

Since the overall coefficient of the parameter represents their influence on COVID spread and daily confirmed cases, we input 0.01 to calculate the overall coefficient as an example. The following calculation illustrates how to calculate the overall coefficient of immigration rate.

$$0.01\times 735.614+{0.01}^{2}\times 616.65+{0.01}^{3}\times \left(-157.74\right)=7.418$$

After calculation, the overall coefficient of the immigration rate is 7.418. However, 7.418 does not mean about 7 more individuals will be infected if the immigration rate increases by 0.01 (1%). As a result, the overall coefficient, 7.418, only represents a quantitative increase in daily confirmed cases. Afterwards, we use the same approach as above to calculate the overall coefficient of the other two parameters, including transmission and waning immunity rate. The overall coefficients show in Table 5.

**Table 5:** The overall coefficients of parameters

Parameter	Overall coefficient
Transmission rate	3.096
Waning immunity rate	7.219
Migrate rate	7.418

According to Table 5, results show that both the waning immunity and migration rate are more important than the transmission rate because of higher overall coefficients.

## Discussion

Most previous studies concentrate on the original or Delta strain, and none investigates the Omicron strain after China reopens its borders. Hence, this research utilizes the Erdos Renyl network, optimized computational model $SEIRS$, and python code to predict the COVID trend. Figures 6, 7, 8 and 9 illustrate the forecast of daily confirmed cases, hospital bed needs, ICU bed needs and cumulative deaths. The overall accuracy of the simulation is 0.959, which shows that the simulation is appropriate and the result is credible. Afterwards, the research uses historical data from the Shanghai government to determine the nine parameters and predict the COVID trend. Figures 10, 11 and 12 demonstrate the detailed results. As shown in Fig. 10, Shanghai will experience two waves of COVID after the first wave. Their peaks will occur in mid-August and mid-December. The maximum daily confirmed cases will be around 88,000 and 340,000, respectively. Figure 11 demonstrates that during the second wave, the hospital bed needs will drop from a peak of 24,000 in mid-August to 8,000 in September. Similarly, the peak of ICU bed demand is also in mid-August, and the maximum demand for ICU bed is 1200. Finally, its needs will decrease to a minimum of 400 in September. However, the third wave is more severe than the second wave. During this time, there will be 74,000 hospital beds and 3,700 ICU beds for COVID patients, which will happen by the end of December or early January. Figure 12 illustrates the cumulative deaths of about 31,500 in the three waves. The second and third waves caused fewer deaths overall, with 4,000 and 10,500 deaths, respectively.

In general, according to Figs. 7, 8 and 9, among the three waves of COVID in 2023, the first wave is the most severe. The remaining outbreaks were far less severe than the first wave. While the second wave of COVID is not expected to result in a medical resources shortage, a more severe medical resources shortage is expected in the third wave. So far, there are 141,000 hospital beds and 1497 ICU beds in Shanghai. Therefore, ICU bed will be in short supply in mid-November due to the third wave of COVID. Fortunately, existing hospital beds are sufficient for the COVID outbreak the following year. According to Fig. 10, with China opening its borders, 31,500 people will die from COVID in Shanghai during the three waves.

According to the above results, they demonstrate that the second and third will occur in 2023. Finding factors that reduce COVID cases daily is crucial to minimise economic and health risks. This research utilises the historical data from the Shanghai government to build the statistical model to investigate the impact of transmission, migration and waning immunity rate on COVID spread and daily confirmed cases increasing. The hypothesis test and p-value demonstrate which statistical model is the most appropriate in this part. Firstly, we determine which variance is more suitable for these data, such as homogeneous and heterogeneous variance. The p-value rejects the null hypothesis. Consequently, the mixed-effect model with heterogeneous variance can demonstrate valuable results. Secondly, the system applies another hypothesis test to decide whether the mixed-effect model is under variances per stratum (varIdent) or power of a covariance (varPower). Since the relatively lower degree of freedom (DF) and lower AIC is our preferred, the mixed-effect model with varPower is the best statical model. After the calculation, the overall coefficient of the transmission, waning immunity, and migration rate are 3.096, 7.219, and 7.418, respectively. Therefore, waning immunity and migration rate are two essential parameters in COVID spread. Decreasing these two parameters can significantly reduce the number of daily confirmed cases, especially migration rate. It is crucial for the government to compile the COVID self-prevention guide for residents after China opened its borders.

## Conclusion

After describing the prior studies, this research utilizes a modified computational model $SEIRS$ and python code to predict the COVID spreading trend, and the medical resources needs after China reopening the border. Moreover, the research also builds statistical models to investigate which parameter significantly impacts COVID daily new cases among transmission, migration, and waning immunity rate. These findings provide a strong basis for the government to prepare medical resources and develop guidelines for citizen self-prevention after China reopens the border.

### Implication

The simulation results indicate that the second and third waves will happen in May–June 2023 and Oct-Dec 2023, respectively. This shows that the outbreak is far from over. Therefore, the government should remind the public not to ignore COVID. For example, people maintain a safe distance from others as far as possible in public places. In the meantime, wearing a mask in crowded places is also a practical approach to self-protection.

Moreover, the government also prepare more medical resource, such as ICU bed. Although the number of hospital beds and ICU beds available is enough for the second wave, there is a shortfall of nearly 2,200 ICU beds in the third wave. Fortunately, the number of hospital beds is sufficient. There are 160,000 hospital beds in Shanghai. The peak of hospital demand is about 81,000. Consequently, preparing more ICU beds before the third wave is the priority for the government.

In this study, we choose Shanghai as the research background because it is representative in China. Hence, the result of this study can demonstrate the situation in China after reopening the border. According to the above result, Shanghai is facing a shortage of medical resources, including ICU beds. Since the number of ICU beds per 100,000 people in Shanghai is 5.99, much higher than the average level in China, 4.6 ICU beds per 100,000 [40]. Therefore, the shortage of ICU beds in the other regions of China will be even more serious. Therefore, the government should prepare more ICU beds before the peak of the third wave.

According to the mixed-effect model built in this study, the result demonstrates that the waning immunity and migration rate are two essential parameters in COVID spreading. Therefore, the government should attach importance to residences’ immunity against COVID because decreasing immunity strength will cause higher infection probability and inflection. Also, before the second wave of the epidemic comes, the government can encourage people to work at home and limit the time of going out to decrease the migration rate, reducing the number of daily confirmed cases. Furthermore, the government should compile and update the COVID self-prevention guide for residents to illustrate which approach is the most appropriate to achieve the most effective self-prevention.

### Limitation

Although we build six networks based on population density and migration characteristics, we do not contain community scenarios like campuses, parks, and supermarkets. The population density of these places is usually high, which may lead to higher transmission rates and more infections. Hence, creating more scenarios in further simulation is an upgrading area of future studies.

Furthermore, the hospitalization, ICU, and mortality rates vary by age group. To illustrate, hospitalization rates for older people are always higher than for younger people. Hence, considering age structure will further improve the accuracy of the simulation, which is another further study.

## Data Availability

The data is from third-party institutions working with local Health Commission, and these data do not permit fully published or shared because of regulations (the data contains sensitive information).

## Abbreviations

ICU: Intensive care unit
ODEs: Ordinary differential equations
PDE: Partial differential equation
DDEs: Delay differential equations
MAE: Mean absolute error
MSE: Mean squared error
LM: Linear model
AIC: Akaike information criterion
DF: Degree of freedom
varIdent: Variances per stratum (varIdent) and power of a covariate
varPower: Variances per stratum (varIdent) and power of a covariate

## References

[1] Wang Y, Fang Z, Gao W (2021). COVID-19’s impact on China’s economy: a prediction model based on railway transportation statistics. Disasters.

[2] Kynge, J. The human and economic cost of China’s zero-Covid strategy is mounting. Financial Times. 2022. Retrieved from https://www.ft.com/content/564be705-2de0-4837-acc6-fe87c3e61aec

[3] National Bureau of Statistics of China. National Economy Withstood Pressure and Reached a New Level in 2022. 2023. Retrieved from www.stats.gov.cn website: http://www.stats.gov.cn/english/PressRelease/202301/t20230117_1892094.html

[4] Mahajan A, Solanki R, Sivadas N (2021). Estimation of undetected symptomatic and asymptomatic cases of COVID-19 infection and prediction of its spread in the USA. J Med Virol.

[5] WHO. Pneumonia of unknown cause – China. 2020. Retrieved from www.who.int website: https://www.who.int/emergencies/disease-outbreak-news/item/2020-DON229

[6] World Health Organization. WHO Statement Regarding Cluster of Pneumonia Cases in Wuhan, China. 2020. Retrieved from www.who.int website: https://www.who.int/china/news/detail/09-01-2020-who-statement-regarding-cluster-of-pneumonia-cases-in-wuhan-china

[7] Katella, K. Omicron, Delta, Alpha, and More: What To Know About the Coronavirus Variants. 2023. Retrieved from Yale Medicine website: https://www.yalemedicine.org/news/covid-19-variants-of-concern-omicron

[8] World Health Organization. Classification of Omicron (B.1.1.529): SARS-CoV-2 Variant of Concern. 2021a. Retrieved from www.who.int website: https://www.who.int/news/item/26-11-2021-classification-of-omicron-(b.1.1.529)-sars-cov-2-variant-of-concern

[9] World Health Organization. Update on Omicron. 2021b. Retrieved from www.who.int website: https://www.who.int/news/item/28-11-2021-update-on-omicron

[10] Tsui WNT, Hamill V, Noll L, Lu N, Porter EP, Harbidge D, Bai J (2022). Molecular detection of SARS-CoV-2 and differentiation of Omicron and Delta variant strains. Transbound Emerg Dis.

[11] Asif A, Ilyas I, Abdullah M, Sarfraz S, Mustafa M, Mahmood A (2022). The comparison of mutational progression in SARS-CoV-2: a short updated overview. J Mol Pathol.

[12] Xu, A., Hong, B., Lou, F., Wang, S., Li, W., Shafqat, A., … Fan, H. Sub‐lineages of the SARS‐CoV‐2 Omicron variants: Characteristics and prevention. MedComm. 2022;3(3). 10.1002/mco2.172 10.1002/mco2.172 PMC9380698 35992968

[13] Liu, L. The dynamics of early-stage transmission of COVID-19: A novel quantification of the role of global temperature. Gondwana Res. 2022;114. 10.1016/j.gr.2021.12.010 10.1016/j.gr.2021.12.010 PMC8747780 35035256

[14] Liu L (2020). Emerging study on the transmission of the Novel Coronavirus (COVID-19) from urban perspective: evidence from China. Cities.

[15] Wang Q, Liu L (2021). On the critical role of human feces and public toilets in the transmission of COVID-19: evidence from China. Sustain Cities Soc.

[16] Aslan IH, Demir M, Wise MM, Lenhart S (2022). Modeling COVID-19: Forecasting and analyzing the dynamics of the outbreaks in Hubei and Turkey. Math Methods Appl Sci.

[17] Boyle L, Hletko S, Huang J, Lee J, Pallod G, Tung H-R, Durrett R (2022). Selective sweeps in SARS-CoV-2 variant competition. Proc Natl Acad Sci.

[18] Wang H, Yamamoto N (2020). Using a partial differential equation with google mobility data to predict COVID-19 in Arizona. Math Biosci Eng.

[19] Gaeta G (2021). A simple SIR model with a large set of asymptomatic infectives. Math Eng.

[20] Dey, S. K., Rahman, Md. M., Shibly, K. H., Siddiqi, U. R., Howlader, A. Epidemic trend analysis of SARS‐CoV‐2 in South Asian Association for Regional Cooperation countries using modified susceptible‐infected‐recovered predictive model. Eng Rep. 2022;5(1). 10.1002/eng2.12550 10.1002/eng2.12550 PMC9349771 35941912

[21] Abdallah W, Kanzari D, Sallami D, Madani K, Ghedira K (2022). A deep reinforcement learning based decision-making approach for avoiding crowd situation within the case of Covid’19 pandemic. Comput Intell.

[22] Trejo I, Hengartner NW (2022). A modified Susceptible-Infected-Recovered model for observed under-reported incidence data. PLOS ONE.

[23] Guglielmi N, Iacomini E, Viguerie A (2022). Delay differential equations for the spatially resolved simulation of epidemics with specific application to COVID-19. Math Methods Appl Sci.

[24] Mahanty, C., Kumar, R., Mishra, B. K., Hemanth, D. J., Gupta, D., Khanna, A. Prediction of COVID‐19 active cases using exponential and non‐linear growth models. Expert Syst. 2020;39(3). 10.1111/exsy.12648

[25] Tiwari D, Bhati BS, Al-Turjman F, Nagpal B (2021). Pandemic coronavirus disease (Covid-19): world effects analysis and prediction using machine-learning techniques. Expert Systems.

[26] Bartolucci F, Pennoni F, Mira A (2021). A multivariate statistical approach to predict COVID-19 count data with epidemiological interpretation and uncertainty quantification. Stat Med.

[27] Wang Y, Zhang Y, Zhang X, Liang H, Li G, Wang X (2022). An intelligent forecast for COVID-19 based on single and multiple features. Int J Intell Syst.

[28] Zhang T, Li J (2021). Understanding and predicting the spatio-temporal spread of COVID-19 via integrating diffusive graph embedding and compartmental models. Trans GIS.

[29] Draief M (2006). Epidemic processes on complex networks. Physica A.

[30] Pastor-Satorras R, Castellano C, Van Mieghem P, Vespignani A (2015). Epidemic processes in complex networks. Rev Mod Phys.

[31] Yang L-X, Deng Y, Piqueira JRC (2017). Epidemic processes on complex networks. Discret Dyn Nat Soc.

[32] Aiello AE, Simanek AM, Eisenberg MC, Walsh AR, Davis B, Volz E, Cheng C, Rainey JJ, Uzicanin A, Gao H, Osgood N, Knowles D, Stanley K, Tarter K, Monto AS (2016). Design and methods of a social network isolation study for reducing respiratory infection transmission: The eX-FLU cluster randomized trial. Epidemics.

[33] Bucur D, Holme P (2020). Beyond ranking nodes: Predicting epidemic outbreak sizes by network centralities. PLOS Comput Biol.

[34] Pollett, S., Johansson, M. A., Reich, N. G., Brett-Major, D., Del Valle, S. Y., Venkatramanan, S., … Morgan, J. J. Recommended reporting items for epidemic forecasting and prediction research: The EPIFORGE 2020 guidelines. PLOS Med. 2021;18(10): e1003793. 10.1371/journal.pmed.1003793 10.1371/journal.pmed.1003793 PMC8525759 34665805

[35] Ioannidis JPA, Zonta F, Levitt M (2023). Estimates of COVID-19 deaths in Mainland China after abandoning zero COVID policy. Eur J Clin Invest.

[36] Heng K, Kitzmann D (2017). The theory of transmission spectra revisited: a semi-analytical method for interpreting WFC3 data and an unresolved challenge. Mon Not R Astron Soc.

[37] Zhang Y, Wei Y, Zhang J (2020). Overpopulation and urban sustainable development—population carrying capacity in Shanghai based on probability-satisfaction evaluation method. Environ Dev Sustain.

[38] Pan, Y., Wang, L., Feng, Z., Xu, H., Li, F., Shen, Y., … Wang, Q. Characterisation of SARS-CoV-2 variants in Beijing during 2022: an epidemiological and phylogenetic analysis. Lancet. 2023. 10.1016/s0140-6736(23)00129-0 10.1016/S0140-6736(23)00129-0 PMC9949854 36773619

[39] Tan L, Wu X, Guo J, Santibanez-Gonzalez EDR (2021). Assessing the Impacts of COVID-19 on the Industrial Sectors and Economy of China. Risk Analy.

[40] Yin H, Wang S, Zhu Y, Zhang R, Ye X, Wei J, Hou PC (2020). The Development of Critical Care Medicine in China: From SARS to COVID-19 Pandemic. Crit Care Res Prac.

